# Palliative resection or radiation of primary tumor prolonged survival for metastatic esophageal cancer

**DOI:** 10.1002/cam4.2609

**Published:** 2019-10-14

**Authors:** Jing Xu, Donghui Lu, Li Zhang, Jian Li, Guoping Sun

**Affiliations:** ^1^ Department of Medical Oncology The First Affiliated Hospital of Anhui Medical University Hefei Anhui China; ^2^ Department of Radiology The 901st Hospital of the Joint Logistics Support Force of PLA Hefei Anhui Province China

**Keywords:** metastatic esophageal cancer, nomogram, palliative resection, radiation, SEER, survival

## Abstract

**Purpose:**

We aimed to explore the value of palliative resection or radiation of primary tumor for metastatic esophageal cancer (EC) patients.

**Methods:**

Surveillance, Epidemiology, and End Results database was used for identifying metastatic EC patients. The patients were divided into resection and nonresection groups. And patients without resection were divided into radiation and nonradiation groups. Propensity score matching (PSM) analyses were adopted to reduce the baseline differences between the groups. Cancer specific survivals (CSSs) and overall survivals (OSs) were compared by Kaplan‐Meier (K‐M) curves. Multivariable analyses by COX proportion hazards model were performed to identify risk factors for CSS and OS. Predictive nomograms were conducted according to both postoperative factors and preoperative factors.

**Results:**

A total of 7982 metastatic EC patients were selected for our analyses. After PSM, 978 patients were included in the survival analyses comparing palliative resection and nonresection. The CSS and OS for patients underwent palliative resection were significantly longer than those without resection (median CSS: 21 months vs 7 months, *P* < .001; median OS: 20 months vs 7 months, *P* < .001). In the overall population without resection, 654 patients were matched for radiation and nonradiation groups. And K‐M curves showed that patients with radiation had longer CSS and OS than those without radiation (median CSS: 11 months vs 6 months, *P* < .001; median OS: 10 months vs 6 months, *P* < .001). Nomograms were generated for prediction of 1‐, 2‐, and 3‐year CSS and OS. All C‐indexes implied moderate discrimination and accuracy. And all nomograms had good calibration.

**Conclusion:**

Palliative resection or radiation of primary tumor could prolong CSS and OS of metastatic EC patients.

## Graphical abstract

This study aimed to evaluate the value of palliative resection or radiation of primary tumor for metastatic esophageal cancer using the Surveillance, Epidemiology, and End Results database. Additionally, we constructed prognostic nomograms for both preoperative and postoperative risk factors. We found that palliative resection or radiation could improve the survival of such patients, across both squamous cell carcinoma and adenocarcinoma.

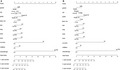

## 1 BACKGROUND

The incidence of esophageal cancer (EC) ranks seventh in all cancer incidence, and it is the sixth cancer‐related death cause worldwide.^1^ More than 30% of patients had metastatic disease at diagnosis. And the cancer specific survival (CSS) of this patient population is poor, with only 3.4% of 5‐year survival.^2^ Managements for metastatic EC patients were usually limited to chemotherapy, endoscopic therapy, and best supportive care. Patients’ clinical performance is the main concern for treatment choices. Local therapies including palliative resection or radiation of primary tumor were only applied to reduce the EC related symptoms (obstruction or bleeding) and improve quality of life for metastatic EC patients.^3^

Previous studies questioned the prognostic value of palliative resection of primary tumor. Tanaka et al investigated 80 metastatic esophageal squamous cell carcinoma patients, and found no difference in survival for palliative resection group and patients without resection.^4^ Saddoughi et al estimated 52 stage IV EC patients with palliative surgery in their institution. The median survival was 10.8 months for these patients, leading to the conclusion that surgery should not be recommended for stage IV EC.^5^ However, some articles revealed better prognosis in patients with stage IV EC after multimodality therapy with palliative resection, and/or radiation, and chemotherapy.^6^, ^7^, ^8^

The Surveillance, Epidemiology, and End Results (SEER) database provides real‐world information on cancer statistics among American population. We aim to explore the value of palliative resection or radiation of tumor in metastatic EC based on the data from SEER database.

## 2 PATIENTS AND METHODS

SEER*Stat version 8.3.5 (with additional treatment from 1975 to 2016) were utilized to identify metastatic EC patients. Palliative resection of primary tumor was defined as cancer‐direct surgery on primary site, not including local tumor destruction (eg, photodynamic therapy, fulguration, cryosurgery, laser, electrocautery, laser, polypectomy, and excisional biopsy). Baseline information and treatments were collected, including age, gender, race, grade, site, histopathological type, AJCC sixth edition TNM stage, tumor size, regional lymph node (LN), distant metastatic organs, CSS months, overall survival (OS), resection or radiation of primary tumor, and chemotherapy. As the wide‐used classification nowadays is the AJCC eighth edition, we translated the sixth edition codes into their corresponding eighth edition codes to generate a uniform dataset.

Patients were excluded if they met the following criteria: (a) Patients died of other causes, not because of EC; (b) Patients’ surgery status was unknown and patients underwent local tumor destruction (eg, photodynamic therapy, fulguration, cryosurgery, laser, electrocautery, laser, polypectomy, and excisional biopsy); (c) The baseline information (eg, race, site, and grade) was not available; (d) The histopathological type was not adenocarcinoma or squamous cell carcinoma.

Chi‐square analyses were performed to detect the statistical differences of each factors between investigated groups. Then we adopted propensity score matching (PSM) analyses to reduce the differences between the groups.^9^ CSSs and OSs of the matched patents were further estimated by Kaplan‐Meier (K‐M) curves. In order to conduct the predictive nomogram, independent risk factors were identified by multivariate analyses. In this study, 70% patients were randomly selected for the training group and the rest were included for validation group. C‐indexes were calculated to discriminate the predictive survival of the nomogram from actual survival. If C‐index was 0.5, the nomogram was supposed to be no discrimination. Nomogram showed perfect discrimination when C‐index was 1.0. Moreover we plotted calibration curves to estimate the accuracy of the nomograms.^10^, ^11^ All *P*‐values less than .05 were considered significant. SPSS 24.0 (SPSS) was employed for Chi‐square analyses, cox regression, and K‐M curves. And Rstudio based on R software 3.5 (Institute for Statistics and Mathematics) was used for nomogram conductions. All *P*‐values were two‐tailed.

## 3 RESULTS

### 3.1 Patients and baseline characteristics

A total of 14 942 metastatic EC patients were retrieved from the SEER database. After excluding patients with incomplete information, 7982 metastatic EC patients were selected for our analyses. Mean age of all population was 63.92 with SD of 11.461. So patients were divided into two age groups: under 63 years old and above 63 years old. The baseline characteristics of these patients are listed in Table 1.

**Table 1:** Characteristics of all metastatic esophageal cancer patients and propensity score‐matching analysis for resection and nonresection groups

Characteristic	All patients			PSM patients		
	Resection (n = 489) No. of patient (%)	Nonresection (n = 7493) No. of patient (%)	*P*	Resection (n = 489) No. of patient (%)	Nonresection (n = 489) No. of patient (%)	*P*
Age			<.001			.645
≤63	307 (62.8%)	3687 (49.2%)		307 (62.8%)	300 (61.3%)	
>63	182 (37.2%)	3806 (50.8%)		182 (37.2%)	189 (38.7%)	
Gender			.068			.322
Male	422 (86.3%)	6228 (83.1%)		422 (86.3%)	411 (84.0%)	
Female	67 (13.7%)	1265 (16.9%)		67 (13.7%)	78 (16.0%)	
Race			.020			.150
White	437 (89.4%)	6349 (84.7%)		437 (89.4%)	428 (87.5%)	
Black	34 (7.0%)	774 (10.3%)		34 (7.0%)	30 (6.1%)	
Other	18 (3.7%)	370 (4.9%)		18 (3.7%)	31 (6.3%)	
Site			<.001			.941
Cervical	2 (0.4%)	78 (1.0%)		2 (0.4%)	2 (0.4%)	
Thoracic	12 (2.5%)	226 (3.0%)		12 (2.5%)	11 (2.2%)	
Abdominal	3 (0.6%)	50 (0.7%)		3 (0.6%)	3 (0.6%)	
Upper third	7 (1.4%)	295 (3.9%)		7 (1.4%)	9 (1.8%)	
Middle third	41 (8.4%)	1033 (13.8%)		41 (8.4%)	35 (7.2%)	
Lower third	407 (83.2%)	5341 (71.3%)		407 (83.2%)	417 (85.3%)	
Overlap	17 (3.5%)	470 (6.3%)		17 (3.5%)	12 (2.5%)	
Grade			.894			.131
Grade I/II	201 (41.1%)	3057 (40.8%)		201 (41.1%)	178 (36.4%)	
Grade III	288 (58.9%)	4436 (59.2%)		288 (58.9%)	311 (63.6%)	
Histopathology			<.001			.933
SCC	86 (17.6%)	2082 (27.8%)		86 (17.6%)	85 (17.4%)	
Adenocarcinoma	403 (82.4%)	5411 (72.2%)		403 (82.4%)	404 (82.6%)	

Abbreviations: lower third, lower third of thoracic esophageal cancer; middle third, middle third of thoracic esophageal cancer; PSM, propensity score‐matched; SCC, squamous cell carcinoma; upper third, upper third of thoracic esophageal cancer.

### 3.2 Survival analyses

**Figure 1:**
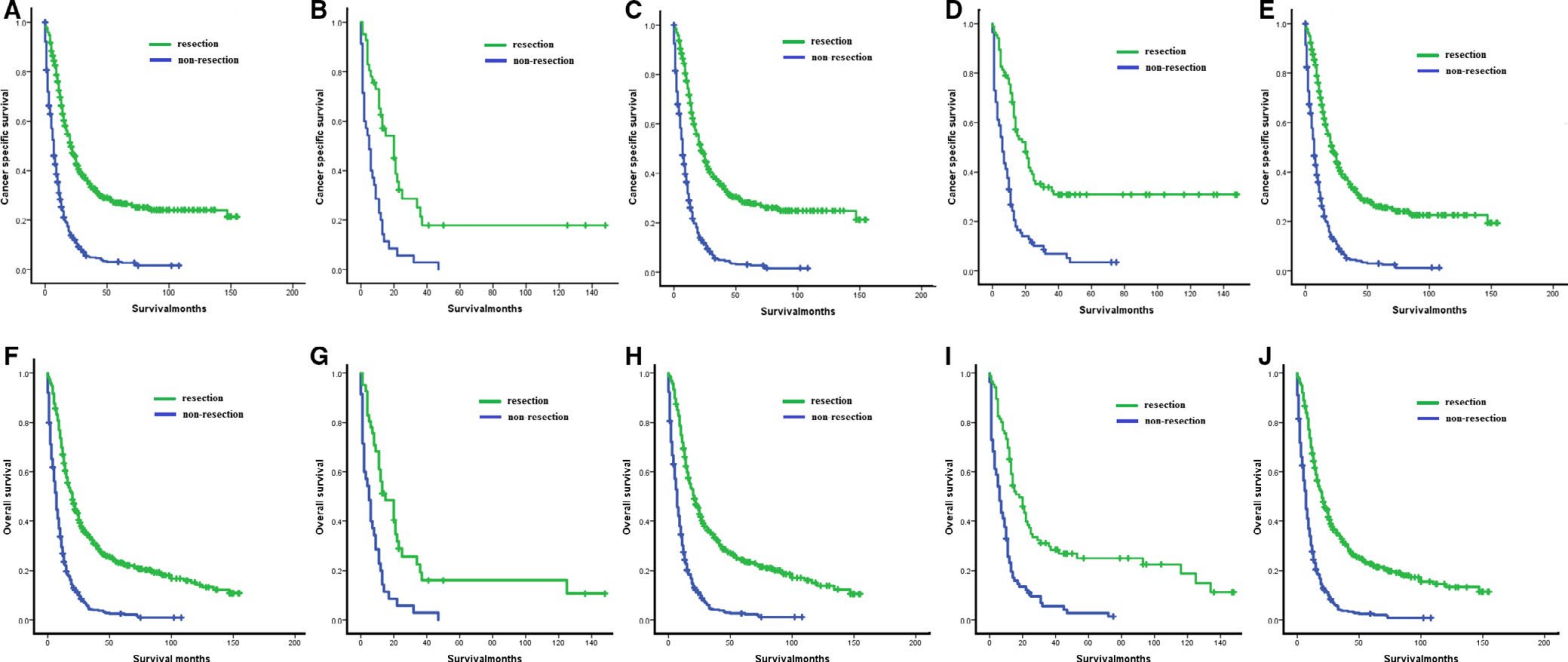
Kaplan‐Meier analyses for survival in different patient cohorts with or without resection. Cancer‐specific survival: A, Overall patients: resection vs nonresection; B, patients with middle third of thoracic esophageal cancer: resection vs nonresection; C, patients with lower third of thoracic esophageal cancer: resection vs nonresection; D, patients with esophageal squamous cell cancer: resection vs nonresection; E, patients with esophageal adenocarcinoma: resection vs nonresection. Overall survival: F, overall patients: resection vs nonresection; G, patients with middle third of thoracic esophageal cancer: resection vs nonresection; H, patients with lower third of thoracic esophageal cancer: resection vs nonresection; I, patients with esophageal squamous cell cancer: resection vs nonresection; J, patients with esophageal adenocarcinoma: resection vs nonresection

Patients with palliative resection and nonresection were matched according to age, race, site, and histopathological type (Table 1). The caliper width of 0.002 was adopted. After PSM, 978 patients were included in the survival analysis comparing palliative resection and nonresection. The CSS and OS for patients underwent palliative resection were significantly longer than those without resection (median CSS: 21 months vs 7 months, *P* < .001, Table 2, Figure 1A; median OS: 20 months vs 7 months, *P* < .001, Table 2, Figure 1F).

**Table 2:** Kaplan‐Meier analyses for survivals in different patient cohorts with or without resection

	Resection		Nonresection		*P*
	No.	Median survival (95% CI)	No.	Median survival (95% CI)	
Overall population	489	CSS: 21 (18.293, 23.707)	489	CSS: 7 (6.077, 7.923)	<.001
		OS: 20 (17.535, 22.465)		OS: 7 (6.234, 7.766)	<.001
Middle third	41	CSS: 20 (13.021, 26.979)	35	CSS: 5 (2.102, 7.898)	<.001
		OS: 15 (7.143, 22.857)		OS: 5 (2.102, 7.898)	<.001
Lower third	407	CSS: 22 (18.791, 25.209)	417	CSS: 7 (6.004, 7.996)	<.001
		OS: 21 (17.828, 24.172)		OS: 7 (6.055, 7.945)	<.001
SCC	86	CSS: 20 (14.376, 25.624)	85	CSS: 6 (3.916, 8.084)	<.001
		OS: 18 (12.579,23.421)		OS: 6 (3.916,8.084)	<.001
Adenocarcinoma	403	CSS: 22 (18.913, 25.087)	404	CSS: 7 (6.005, 7.995)	<.001
		OS: 20 (16.694, 23.306)		OS: 7 (6.170, 7.830)	<.001

Abbreviations: CSS, cancer‐specific survival; lower third, lower third of thoracic esophageal cancer; middle third, middle third of thoracic esophageal cancer; No., number of patients; OS, overall survival; SCC, squamous cell carcinoma; 95% CI, 95% confidence interval.

For subgroup analyses, patients with palliative resection still live longer than those without resection (Table 2). Patients with middle‐ and lower‐third of thoracic EC could benefit from palliative resection (middle‐third CSS: 20 vs 5 months, Figure 1B; middle‐third OS: 15 vs 5 months, Figure 1G; lower‐third CSS: 22 vs 7 months, Figure 1C; lower‐third OS: 21 months vs 7 months, Figure 1H). This trend remained for patients with squamous cell carcinoma, as palliative resection led to 20 months CSS and 18 months OS, and patients without resection had 6 months CSS and 6 months OS (Figure 1D,I). Moreover 807 patients were diagnosed with adenocarcinoma. And K‐M analysis showed that patients with palliative resection had median CSS of 22 months and median OS of 20 months, while patients without resection only had median CSS and OS of 7 months, respectively (Figure 1E,J).

**Figure 2:**
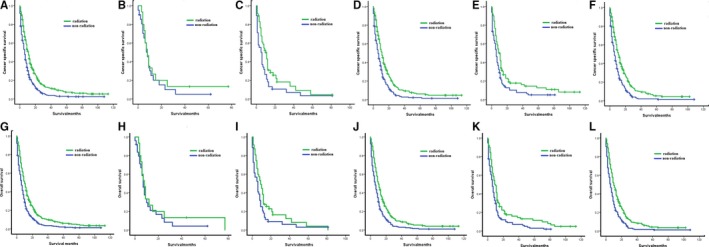
Kaplan‐Meier analyses for survival in different patient cohorts with or without radiation. Cancer‐specific survival: A, Overall patients: radiation vs nonradiation; B, patients with upper third of thoracic esophageal cancer: radiation vs nonradiation; C, patients with middle third of thoracic esophageal cancer: radiation vs nonradiation; D, patients with lower third of thoracic esophageal cancer: radiation vs nonradiation; E, patients with esophageal squamous cell cancer: radiation vs nonradiation; F, patients with esophageal adenocarcinoma: radiation vs nonradiation. Overall survival: G, Overall patients: radiation vs nonradiation; H, patients with upper third of thoracic esophageal cancer: radiation vs nonradiation; I, patients with middle third of thoracic esophageal cancer: radiation vs nonradiation; J, patients with lower third of thoracic esophageal cancer: radiation vs nonradiation; K, patients with esophageal squamous cell cancer: radiation vs nonradiation; L, patients with esophageal adenocarcinoma: radiation vs nonradiation

In the overall population without resection, 654 patients were matched for radiation and nonradiation groups with a caliper width of 0.001 (Table 3). And K‐M curves indicated that patients with radiation had longer CSS and OS than those without radiation (median CSS: 11 months vs 6 months, *P* < .001, Table 4, Figure 2A; median OS: 10 months vs 6 months, *P* < .001, Table 4, Figure 2G).

**Table 3:** Characteristics of patients without resection and propensity score‐matching analysis for radiation and nonradiation groups

Characteristic	Nonresection			PSM patients		
	Radiation (n = 327) No. of patient (%)	Nonradiation (n = 7166) No. of patient (%)	*P*	Radiation (n = 327) No. of patient (%)	Nonradiation (n = 327) No. of patient (%)	*P*
Age			.023			.875
≤63	181 (55.4%)	3506 (48.9%)		181 (55.4%)	183 (56.0%)	
>63	146 (44.6%)	3660 (51.1%)		146 (44.6%)	144 (44.0%)	
Gender			.432			.400
Male	277 (84.7%)	5951 (83.0%)		277 (84.7%)	269 (82.3%)	
Female	50 (15.3%)	1215 (17.0%)		50 (15.3%)	58 (17.7%)	
Race			.098			1.000
White	280 (85.6%)	6069 (84.7%)		280 (85.6%)	280 (85.6%)	
Black	25 (7.6%)	749 (10.5%)		25 (7.6%)	25 (7.6%)	
Other	22 (6.7%)	348 (4.9%)		22 (6.7%)	22 (6.7%)	
Site			.396			.203
Cervical	4 (1.2%)	74 (1.0%)		4 (1.2%)	1 (0.3%)	
Thoracic	13 (4.0%)	213 (3.0%)		13 (4.0%)	6 (1.8%)	
Abdominal	2 (0.6%)	48 (0.7%)		2 (0.6%)	2 (0.6%)	
Upper third	15 (4.6%)	280 (3.9%)		15 (4.6%)	21 (6.4%)	
Middle third	47 (14.4%)	986 (13.8%)		47 (14.4%)	38 (11.6%)	
Lower third	235 (71.9%)	5106 (71.3%)		235 (71.9%)	240 (73.4%)	
Overlap	11 (3.4%)	459 (6.4%)		11 (3.4%)	19 (5.8%)	
Grade			.032			.875
Grade I/II	152 (46.5%)	2905 (40.5%)		152 (46.5%)	150 (45.9%)	
Grade III	175 (53.5%)	4261 (59.5%)		175 (53.5%)	177 (54.1%)	
Histopathology			.914			.728
SCC	90 (27.5%)	1992 (27.8%)		90 (27.5%)	94 (28.7%)	
Adenocarcinoma	237 (72.5%)	5174 (72.2%)		237 (72.5%)	233 (71.3%)	

Abbreviations: lower third, lower third of thoracic esophageal cancer; middle third, middle third of thoracic esophageal cancer; PSM, propensity score‐matched; SCC, squamous cell carcinoma; upper third, upper third of thoracic esophageal cancer.

**Table 4:** Kaplan‐Meier analyses for survivals in different patient cohorts with or without radiation

	Radiation		Nonradiation		*P*
	No.	Median survival (95% CI)	No.	Median survival (95% CI)	
Overall population	327	CSS: 11 (9.596, 12.404)	327	CSS: 6 (5.064, 6.936)	<.001
		OS: 10 (8.637, 11.363)		OS: 6 (5.055, 6.945)	<.001
Upper third	15	CSS: 8 (5.160, 10.840)	21	CSS: 8 (5.395, 10.605)	.539
		OS: 8 (5.160, 10.840)		OS: 7 (5.092, 8.908)	.454
Middle third	47	CSS: 11 (7.368, 14.632)	38	CSS: 5 (2.411, 7.589)	.023
		OS: 9 (5.641, 12.359)		OS: 6 (3.260, 8.740)	.045
Lower third	235	CSS: 11 (9.451, 12.549)	240	CSS: 6 (4.788, 7.212)	<.001
		OS: 10 (8.233, 11.767)		OS: 6 (4.689, 7.311)	<.001
SCC	90	CSS: 9 (6.547, 11.453)	94	CSS: 6 (4.518, 7.482)	.005
		OS: 9 (6.676, 11.324)		OS: 6 (4.628, 7.372)	.002
Adenocarcinoma	237	CSS: 11 (9.349, 12.651)	233	CSS: 6 (4.806, 7.194)	<.001
		OS: 10 (8.115, 11.885)		OS: 6 (4.771, 7.229)	<.001

Abbreviations: CSS, cancer‐specific survival; lower third, lower third of thoracic esophageal cancer; middle third, middle third of thoracic esophageal cancer; No., number of patients; OS, overall survival; SCC, squamous cell carcinoma; 95% CI, 95% confidence interval; upper third, upper third of thoracic esophageal cancer.

Table 4 presented the median survivals for subgroup analyses. There was no difference of CSS and OS between radiation group and nonradiation group if the primary tumor was in upper‐third of thoracic esophagus (CSS: *P* = .539, Figure 2B; OS: *P* = .454, Figure 2H). However, radiation benefited patients with middle‐ and lower‐third of thoracic EC (radiation vs nonradiation: CSS of middle‐third was 11 vs 5 months, *P* = .023, Figure 2C, OS of middle‐third was 9 vs 6 months, *P* = .045, Figure 2I; CSS of lower‐third was 11 vs 6 months, *P* < .001, Figure 2D; OS of lower‐third was 10 vs 6 months, *P* < .001, Figure 2J). The survival outcome of patient receiving radiation was also significantly better than those without radiation for squamous cell carcinoma subgroup (median CSS: 9 months vs 6 months, *P* = .005, Figure 2E; median OS: 9 months vs 6 months, *P* = .002, Figure 2K). Furthermore, for patients diagnosed as adenocarcinoma, radiation led to longer CSS and OS compared with nonradiation (median CSS: 11 months vs 6 months, *P* < .001, Figure 2F; median OS: 10 months vs 6 months, *P* < .001, Figure 2L).

### 3.3 Nomograms based on preoperative or postoperative risk factors

**Figure 3:**
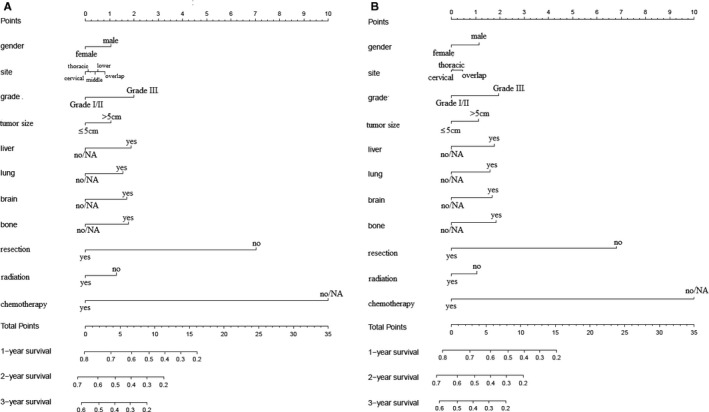
Preoperative nomograms. A, Cancer specific survival: training group; B, overall survival: training group. NA, not available

**Figure 4:**
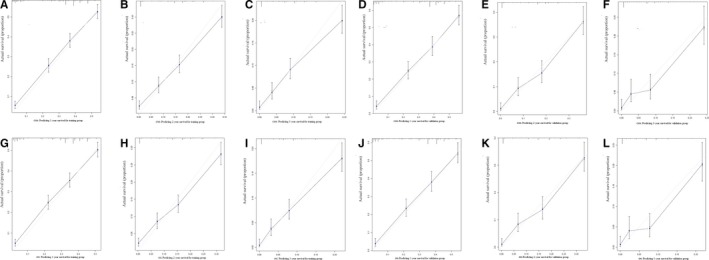
Calibration curves for survival prediction of preoperative nomograms: 1‐y (A), 2‐y (B), and 3‐y (C) of cancer‐specific survival (CSS) for the training group, calibration curves for the CSS prediction at 1 y (D), 2‐y (E), and 3‐y (F) in the validation group, 1‐y (G), 2‐y (H), and 3‐y (I) of overall survival (OS) for the training group, calibration curves for the OS prediction at 1 y (J), 2‐y (K), and 3‐y (L) in the validation group

A total of 4206 patients had detailed preoperative information, such as age, gender, site, grade, tumor size, LN metastasis, distant organ metastases, and treatment choices. Multivariable analyses revealed that gender, site, grade, tumor size, distant metastases, and treatment choices were independently associated with CSS and OS (Table 5). Thus, these risk factors were included for preoperative nomogram. After randomization (ratio: 7:3), 1249 patients were selected for training group and the rest for validation group. The nomograms for CSS and OS prediction based on training group are presented in Figure 3. And the nomograms based on validation group are presented in Figure S1. C‐indexes for CSS prediction were 0.706 and 0.723, respectively, for training and validation groups. And C‐indexes for OS prediction were 0.703 and 0.721, respectively, for training and validation groups. Calibration curves showed good agreement between observed survivals (CSSs and OSs) and predicted survivals from nomograms (Figure 4).

**Table 5:** Multivariable analyses of potential preoperative risk factors for survival

Characteristic	All patients				
	No. of patient	CSS: HR (95% CI)	*P*	OS: HR (95% CI)	*P*
Age			.239		.079
≤63	2104	1		1	
>63	2100	1.041 (0.974, 1.112)		1.060 (0.993, 1.131)	
Gender			<.001		<.001
Male	3535	1		1	
Female	669	0.838 (0.763, 0.919)		0.831 (0.760, 0.910)	
Race			.921		.926
White	3513	1		1	
Black	432	1.018 (0.904, 1.146)		1.023 (0.912, 1.149)	
Other	259	1.023 (0.892, 1.173)		1.005 (0.879, 1.150)	
Site			.004		.003
Cervical	41	1		1	
Thoracic	148	1.154 (0.787, 1.694)		1.091 (0.761, 1.565)	
Abdominal	29	1.289 (0.768, 2.165)		1.268 (0.778, 2.066)	
Upper third	164	1.514 (1.043, 2.198)		1.371 (0.964, 1.949)	
Middle third	606	1.400 (0.987, 1.985)		1.314 (0.947, 1.822)	
Lower third	2953	1.235 (0.873, 1.747)		1.133 (0.819, 1.568)	
Overlap	263	1.520 (1.054, 2.190)		1.404 (0.995, 1.979)	
Grade			<.001		<.001
Grade I/II	1751	1		1	
Grade III	2453	1.225 (1.147, 1.309)		1.212 (1.136, 1.293)	
Histopathology			.961		.985
SCC	1255	1		1	
Adenocarcinoma	2949	0.998 (0.908, 1.096)		1.001 (0.913, 1.097)	
Tumor size			.001		<.001
≤5 cm	2058	1		1	
>5 cm	2146	1.113 (1.042, 1.188)		1.122 (1.052, 1.196)	
LN			.112		.141
Negative	963	1		1	
Positive	3241	0.939 (0.869, 1.015)		0.945 (0.876, 1.019)	
Liver			<.001		<.001
No/NA	3264	1		1	
Yes	940	1.279 (1.180, 1.387)		1.269 (1.172, 1.374)	
Lung			.004		.002
No/NA	3600	1		1	
Yes	604	1.148 (1.045, 1.262)		1.160 (1.058, 1.272)	
Bone			<.001		<.001
No/NA	3720	1		1	
Yes	484	1.294 (1.168, 1.433)		1.301 (1.178, 1.437)	
Brain			.042		.038
No/NA	4084	1		1	
Yes	120	1.222 (1.007, 1.481)		1.221 (1.011, 1.474)	
Resection			<.001		<.001
No	3795	1		1	
Yes	409	0.471 (0.407,0.546)		0.489 (0.424, 0.563)	
Radiation			.003		.009
No	3708	1		1	
Yes	496	0.818 (0.717, 0.934)		0.844 (0.744, 0.958)	
Chemotherapy			<.001		<.001
No/NA	1248	1		1	
Yes	2956	0.316 (0.294, 0.341)		0.322 (0.299, 0.346)	

Abbreviations: CSS, cancer‐specific survival; HR, hazard ratio; LN, regional lymph node; lower third, lower third of thoracic esophageal cancer; middle third, middle third of thoracic esophageal cancer; NA, not available; No., number; OS, overall survival; SCC, squamous cell carcinoma; upper third, upper third of thoracic esophageal cancer; 95% CI, 95% confidence interval.

**Figure 5:**
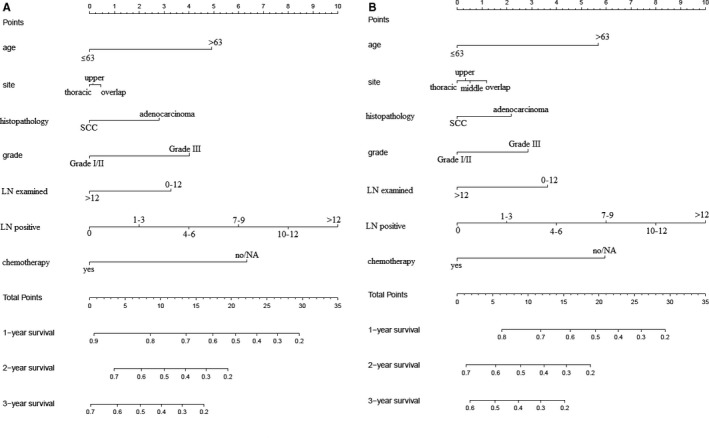
Postoperative nomograms. A, Cancer‐specific survival: training group; B, overall survival: training group. LN, regional lymph node; NA, not available; SCC, squamous cell carcinoma

**Figure 6:**
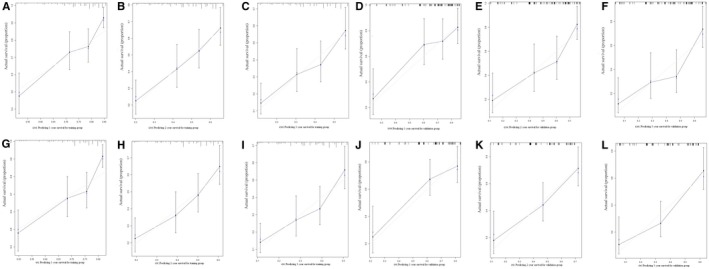
Calibration curves for survival prediction of postoperative nomogram: 1‐y (A), 2‐y (B), and 3‐y (C) of cancer‐specific survival (CSS) for the training group, and calibration curves for the CSS prediction at 1 y (D), 2‐y (E), and 3‐y (F) in the validation group, 1‐y (G), 2‐y (H), and 3‐y (I) of overall survival (OS) for the training group, and calibration curves for the OS prediction at 1 y (J), 2‐y (K), and 3‐y (L) in the validation group

To predicting CSS and OS after palliative resection, we performed nomogram based on postoperative factors. After exclusion of non‐available data, 413 patients who underwent palliative resection were identified for multivariable cox regression (Table 6). It was implied that age, site, histopathological type, grade, LN examined, LN positive, and chemotherapy could independently influence CSS and OS. Randomized selection of 70% radio was utilized to pick out patients for training group. And 291 patients were finally included for training group, and 122 patients for validation group. Postoperative nomograms were generated for CSS and OS in training groups (Figure 5). And nomograms for validation group were shown in Figure S2. C‐indexes indicated moderate discrimination (0.669 and 0.720 for CSS in training and validation groups, respectively; 0.66 and 0.713 for OS in training and validation groups, respectively). Figure 6 showed that these nomograms had good calibration.

**Table 6:** Multivariable analyses of potential postoperative risk factors for survival

Characteristic	Patients with palliative resection				
	No. of patient	CSS: HR (95% CI)	*P*	OS: HR (95%CI)	*P*
Age			.002		.001
≤63	260	1		1	
>63	153	1.500 (1.158, 1.942)		1.507 (1.183, 1.920)	
Gender			.174		.177
Male	356	1		1	
Female	57	0.752 (0.498, 1.134)		0.769 (0.525, 1.126)	
Race			.486		.370
White	368	1		1	
Black	29	1.125 (0.669, 1.892)		1.261 (0.778, 2.044)	
Other	16	0.672 (0.326, 1.384)		0.712 (0.359, 1.412)	
Site			.023		.026
Cervical	10	1		1	
Thoracic	3	0.727 (0.138, 3.843)		0.745 (0.143, 3.895)	
Abdominal	5	1.878 (0.514, 6.863)		1.561 (0.429, 5.677)	
Upper third	34	1.444 (0.578, 3.604)		1.588 (0.650, 3.877)	
Middle third	349	0.752 (0.330, 1.716)		0.847 (0.375, 1.912)	
Lower third	NA	NA		NA	
Overlap	12	1.974 (0.664, 5.866)		2.210 (0.767, 6.368)	
Grade			.003		.020
Grade I/II	176	1		1	
Grade III	237	1.490 (1.144, 1.940)		1.336 (1.047, 1.705)	
Histopathology			.031		.022
SCC	70	1		1	
Adenocarcinoma	343	1.633 (1.045, 2.552)		1.622 (1.072, 2.455)	
Tstage			.723		.246
T1/2	74	1		1	
T3/4	339	1.061 (0.764, 1.474)		1.206 (0.879, 1.654)	
Nstage			.963		.860
N0	133	1		1	
N1/2/3	280	0.993 (0.750, 1.316)		1.024 (0.785, 1.336)	
LN examined			.016		.007
0‐12	185	1		1	
>12	228	0.729 (0.563, 0.942)		0.718 (0.565, 0.913)	
LN positive			<.001		<.001
0	142	1		1	
1‐3	144	1.518 (1.105, 2.085)		1.394 (1.042, 1.866)	
4‐6	61	1.807 (1.214, 2.689)		1.566 (1.079, 2.275)	
7‐9	32	1.731 (1.046, 2.866)		1.412 (0.871, 2.291)	
10‐12	11	1.669 (0.810, 3.439)		1.569 (0.791, 3.112)	
>12	23	4.210 (2.407, 7.364)		3.801 (2.227, 6.489)	
Radiation			.168		.099
No	108	1		1	
Yes	305	0.787 (0.560, 1.106)		0.763 (0.554, 1.052)	
Chemotherapy			.003		.004
No/NA	62	1		1	
Yes	351	0.530 (0.349, 0.804)		0.562 (0.378, 0.835)	

Abbreviations: CSS, cancer‐specific survival; HR, hazard ratio; LN, regional lymph node; lower third, lower third of thoracic esophageal cancer; middle third, middle third of thoracic esophageal cancer; NA, not available; No., number; OS, overall survival; SCC, squamous cell carcinoma; upper third, upper third of thoracic esophageal cancer; 95% CI, 95% confidence interval.

## 4 DISCUSSION

Patients with metastatic EC were not usually recommended palliative resection on primary tumor or radiation by guidelines. Only systemic therapy, palliative supportive care, and sometimes clinical trials were preferred for these patients. The role of palliative resection and radiation is not clear for metastatic EC. However, recent studies have indicated that palliative resection or radiation might benefit for survival.^6^, ^7^, ^8^ Some case reports revealed promising results on long term survival after palliative surgery as well as radiation.^12^, ^13^, ^14^, ^15^, ^16^, ^17^, ^18^ Our results were in accordance with them that patients underwent palliative resection or radiation had prolonged CSS and OS. As we extracted data from SEER database, our results were supposed to reflect the true outcomes of cancer patients in real world.

Palliative resection and radiation are both local treatment for primary tumor. The median CSS and OS of patients receiving palliative resection were 21 months and 20 months, respectively, which was almost 10 months longer than those with radiation. Interestingly, radiation was not an independent predictor after surgery from our multivariable analyses. These results might be due to the reasons that patients selected for surgery had better performance than palliative radiation, and patients with metastatic disease could hardly bear both postoperative and radiation complications. Ando et al suggested that advances in surgical technique and perioperative management improved survival of advanced EC patients.^19^ As SEER database did not provide information about patients’ performance, future studies comparing palliative resection and radiation should be carried out taking this factor into account.

To the best of our knowledge, our study was the first one developing predictive nomograms for metastatic EC. The features at diagnosis, such as gender, site, grade, tumor size, metastatic distant organs, and treatment choices had independent value for predicting survival. However, age and regional LN metastases did not influence the outcome of patients at this point. For the patients with palliative resection, older age, adenocarcinoma, Grade III tumor, examined LNs less than 12, more positive LNs, and nonchemotherapy were factors for poor prognosis. Nevertheless, T stages and N stages were not independent predictor for survival. In routine clinical practice, TNM staging was commonly used for predicting survival of cancer patients. Our nomograms were supposed to be an important supplement for TNM staging and to help select best anticancer therapy for metastatic EC patients at diagnosis.

Palliative resection of primary tumor was suggested for stage IV incurable gastric cancer 20, 21 and colorectal cancer patients.^22^, ^23^ It was implied that patency of digestive system was important for patients’ quality of life. And palliative resection for highly selected patients could prevent tumor related complications such as obstruction, bleeding, and perforation. For EC, the main symptom was dysphagia. And tumor might also cause bleeding and perforation. Palliative resection or radiation removed the primary tumor, reducing the potential risk of serious tumor‐related complications. However, whether the EC patients received emergency or selected surgery was not known from SEER database. Postoperative complication might also be an important risk factor for cancer patients. Future prospective studies should be performed to estimate the postoperative mortality for both surgery types.

Our study had strength in large sample of EC patients and sufficient statistical analyses. These resulted in reliable conclusions. Nevertheless, some shortages still existed. As mentioned previously, the SEER database did not provide information of performance, basic diseases, and surgery type. Although we took chemotherapy into consideration for nomograms, different regimens might lead to various outcomes. And as new treatments, for example, immunotherapies, have emerged, the survival prediction of cancer patient could be more challenging with different combination of treatments.

## 5 CONCLUSION

Metastatic EC patients had prolonged survival with palliative resection or radiation of primary tumor. Our nomograms will aid in selecting dominant crowd for palliative resection.

## CONFLICT OF INTEREST

The authors declare no conflict of interest.

## AUTHOR CONTRIBUTIONS

Jing Xu contributed to conceptualization, methodology, data analyses, writing original draft, writing review and editing, supervision, and project administration. Donghui Lu contributed to methodology, data analyses, validation, writing review and editing, and project administration. Li Zhang contributed to investigation, resources, data analyses, editing, supervision, and project administration. Jian Li contributed to software, validation, data analyses, data curation, writing original draft, and project administration. Guoping Sun contributed to conceptualization, methodology, validation, resources, writing review and editing, visualization, supervision, project administration, and funding acquisition.
