# Supplementary figures and images for: Palliative resection or radiation of primary tumor prolonged survival for metastatic esophageal cancer
Source: Cancer Med. 2019 Oct 14;8(17):7253–64. doi: 10.1002/cam4.2609 (PMC6885868; doi:10.1002/cam4.2609)

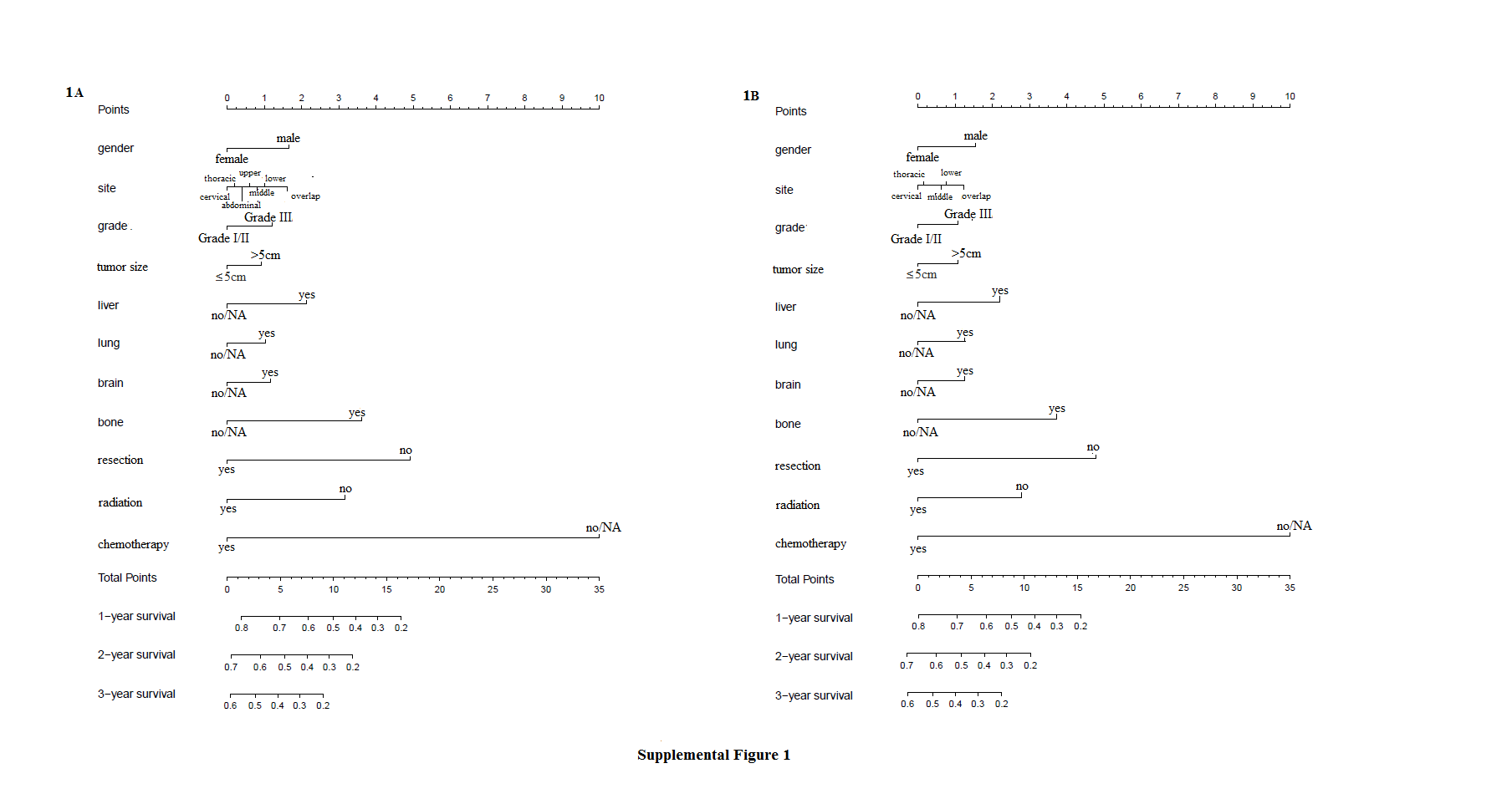

Supplement: Supplementary file 1 [file CAM4-8-7253-s001.tif]

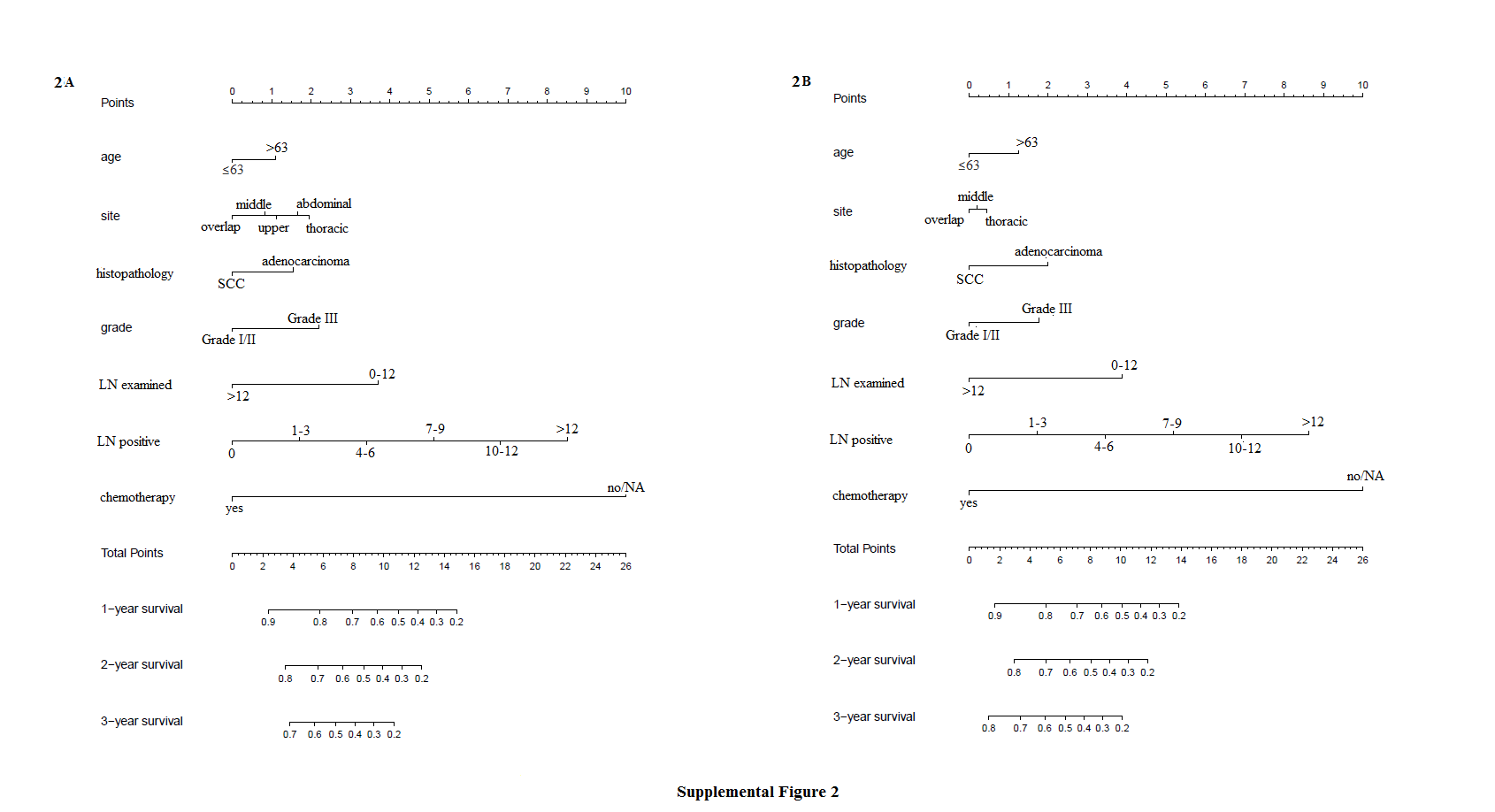

Supplement: Supplementary file 2 [file CAM4-8-7253-s002.tif]
