# Supplementary material for: Palliative resection or radiation of primary tumor prolonged survival for metastatic esophageal cancer
Source: Cancer Med. 2019 Oct 14;8(17):7253–64. doi: 10.1002/cam4.2609 (PMC6885868; doi:10.1002/cam4.2609)
Supplement: Supplementary file 3 [file CAM4-8-7253-s003.docx]

**Supplementary figure legends**

**Supplementary Figure 1.** Preoperative nomograms.

**(3A)** cancer specific survival: validation group, **(3B)** overall survival: validation group

NA: not available

**Supplementary Figure 2.** Postoperative nomograms.

**(2A)** cancer specific survival: validation group, **(2B)** overall survival: validation group

SCC: squamous cell carcinoma, LN: regional lymph node, NA: not available
